# Prediction of monthly evapotranspiration by artificial neural network model development with Levenberg–Marquardt method in Elazig, Turkey

**DOI:** 10.1007/s11356-024-32464-1

**Published:** 2024-02-21

**Authors:** Veysi Kartal

**Affiliations:** Department of Civil Engineering, Siirt University, Siirt, 56000 Turkey

**Keywords:** Estimation, Evapotranspiration, ANN, Levenberg–Marquardt, Machine learning

## Abstract

The phenomenon of evapotranspiration (ET) is closely linked to the issue of water scarcity, as it involves water loss through both evaporation and plant transpiration. Accurate prediction of evapotranspiration is of utmost importance in the strategic planning of agricultural irrigation, effective management of water resources, and precise hydrological modeling. The current investigation aims to predict the monthly ET values in the Elazig province by developing an artificial neural network (ANN) model utilizing the Levenberg–Marquardt method. Consequently, the values of temperature, precipitation, relative humidity, solar hour, and mean wind speed were utilized in forecasting evapotranspiration values by implementing ANN algorithms. This research makes a valuable contribution to the existing body of literature by utilizing an ANN model developed with the Levenberg–Marquardt method to estimate evapotranspiration. It has been discovered that evapotranspiration values are impacted by various factors such as temperature (minimum, average, maximum), relative humidity (minimum, average, maximum), wind speed, solar hour, and precipitation values, which are taken into consideration for prediction. The findings indicated that Elazig, Keban, Baskil, and Agin sites had *R* values of 0.9995, 0.9948, 0.9898, and 0.9994 in the proposed model. It was found that Elazig’s MAPE ranged from 0 to 0.2288, Keban’s was 0.0001 to 0.3703, Baskil’s was between 0 and 0.4453, and Agin’s was both 0 and 0.2784. The findings obtained from the proposed model are compatible with evapotranspiration values computed from the Hargreaves method (*R*^2^ = 0.996). The study’s findings provide significant insights for planners and decision-makers involved in the planning and managing water resources and agricultural irrigation.

## Introduction

Evapotranspiration (ET) represents water scarcity due to evaporation from the plant and soil surfaces and transpiration of vegetation. Evapotranspiration values are very effective in plant management and developing water resource climate and atmospheric circulation (Ding et al. 2010; Huang et al. 2019). ET is crucial for water resource planning, irrigation scheduling, hydrological modeling, agricultural production, and efficient use of water resources (Feng et al. 2016). Evaporation changes according to air temperature, atmospheric pressure, solar radiation, relative humidity, and wind speed (Vicente-Serrano et al. 2018). So, the computation of ET values is regarded as a non-linear and complex process (Fan et al. 2018a). Several hydro-meteorological studies were conducted to solve and investigate the complex relationship using artificial intelligence (AI) models (Shiri et al. 2015; Fotovatikhah et al. 2018; Qasem et al. 2019). Machine learning and different models were applied in different disciplines (Jafari and Jafari 2015; Rahmani et al. 2017; Davarikhah et al. 2020; Behroozpour et al. 2021). Recently, AI techniques have gained popularity because of their ability to link many variables in an easy and fast way (Esfandyari et al. 2018; Davarikhah et al. 2020; Hedayati et al. 2023; Jafari et al. 2023; Borj et al. 2024).

Furthermore, the hydro-meteorological forecasting process has drawn attention due to these features (Huang et al. 2010). With artificial intelligence techniques, it has become common to predict the ET value, which is one of the fundamental elements of hydro-meteorological phenomena. Hargreaves and Samani (HS) model is appropriate for ET prediction in semi-arid climates, while models based on radiation are preferred for humid environments (Kumar et al. 2012). Tabari et al. (2012) compared adaptive neuro-fuzzy inference systems (ANFIS), support vector machine (SVM), multiple non-linear regression (MNLR), multiple linear regression (MLR), and empirical models for prediction of ET in Iran. The results show that SVM and ANFIS present better results than other methods.

Similarly, Wen et al. (2015) show that the results of the SVM model were better than that of artificial neural network and empirical models in the estimation of ET for regions of China with extremely arid while Djaman et al. (2015) stated that mass transfer models were more accurate for the Senegal River Valley. Regarding this issue, Ali Ghorbani et al. (2018) stated that the main disadvantage of empirical models is the lack of consistency, as their accuracy is highly dependent on local calibrations and climatic conditions. Based on the studies of Cobaner et al. (2017) and Gafurov et al. (2018), the simple empirical models lack the universal approach required for ET forecasting. Citakoglu et al. (2014) compared the performance of ANN and ANFIS in estimating the ET values in Turkey. Kisi and Cimen (2009) initiated this when they compared SVM with several empirical models in California, such as the Penman, Ritchie, and Turc models. The study demonstrated that the SVM shows excellent potential to serve as an effective alternative to such empirical models. Shrestha and Shukla (2015) adopted a similar approach. The authors developed Kisi and Cimen’s (2009) study further by using SVM to forecast pepper and watermelon crop coefficients. Although other soft computing techniques have been developed, the performance of SVM is always comparable to that of another machine learning tool. For example, Tabari et al. (2012) investigated the performance of SVM compared to ANFIS for ET prediction in a semi-arid environment. SVM was compared with ANN models to predict ET in Brazil with limited meteorological parameters (Wen et al. 2015; Ferreira et al. 2019).

Feng et al. (2016) implemented genetic algorithm–optimized backpropagation neural networks (GANN), wavelet neural networks (WNN), and trained extreme learning machine (ELM) models to predict ET in Southwest China. They stated that the results of the GANN and ELM models were better than the WNN model. Fan et al. (2018b) used the M5 model tree (M5Tree), ELM, SVM, random forest (RF), extreme gradient boost (XGBoost), and gradient boost decision tree (GBDT) methods in China for the prediction of daily ET values. Based on the study, GBDT and XGBoost algorithms showed the best performance. ET values in Ankara and Kirikkale were predicted by Kisi and Alizamir (2018) using various machine learning models and wavelet rotation. They stated that the wavelet models are more useful than traditional machine learning (ML) models. Huang et al. (2019) investigated the success of SVM, RF, and categorical boosting (CatBoost) methods to forecast daily ET values in China. They determined that all methods present good results in subtropical China, but the CatBoost method significantly improves ET forecasting. The literature shows that numerous meteorological and hydrological prediction models use the ANN and ML methods (Kisi and Alizamir 2018; Chong et al. 2020; Hameed et al. 2021; Quilty and Adamowski 2021; Sarıgöl and Katipoğlu 2023). Katipoglu (2023) predicted ET values by combining discrete wavelet decomposition and soft computing techniques in the semi-arid Hakkâri province. As seen in the literature, it was understood that studies about forecasting using ANN model development with the Levenberg–Marquardt method are limited. The present study will be the literature’s touchstone for assessing the effects of precipitation, solar hour, temperature, humidity, and wind speed on ET prediction. The study’s novelty was ET prediction using ANN model development with the Levenberg–Marquardt method using temperature (min, mean, and max), relative humidity (min, mean, and max), solar hour, mean wind speed, and rainfall. This study aims to evaluate the performance of the Levenberg–Marquardt method in predicting monthly ET in Elazig. For this purpose, nine inputs (temperature (min, mean and max), relative humidity (min, mean and max), mean wind speed, solar hour, and rainfall values) and one output (evapotranspiration) with ANN model were formed to set the model. In addition, the performance of estimating monthly ET values was checked and evaluated using statistical criteria. Although there are studies with many different ANN models in the literature, it has been shown by Sahin et al. (2023), Güzel and Çolak (2023), and Shafiq et al. (2023) that the Levenberg–Marquardt ANN method is superior to other ANN models and correlations in terms of convergence success and precision, and Levenberg–Marquardt method is preferred in this study.

## Study area and data

Elazig province is located southwest of the Eastern Anatolia Region in the Upper Euphrates Region. With an area of 9153 km^2^, it has 0.12% of Turkey’s territory. Elazig is located between 40 0 21′ and 38 0 30′ east longitudes and 38 0 17′ and 39 0 11′ north latitudes. Bingöl surrounds the province from the east, Tunceli from the north (via Keban Dam Lake), Malatya from the west and southwest (via Karakaya Dam Lake), and Diyarbakir from the south. Relative humidity (maximum, average, and minimum), temperature (maximum, average, and minimum), solar hour, evapotranspiration, mean wind speed, and precipitation values of four meteorological stations between 1980 and 2022 were used in the study (Agin, Baskil, Elazig and Keban).

### Data analysis

**Fig. 1:**
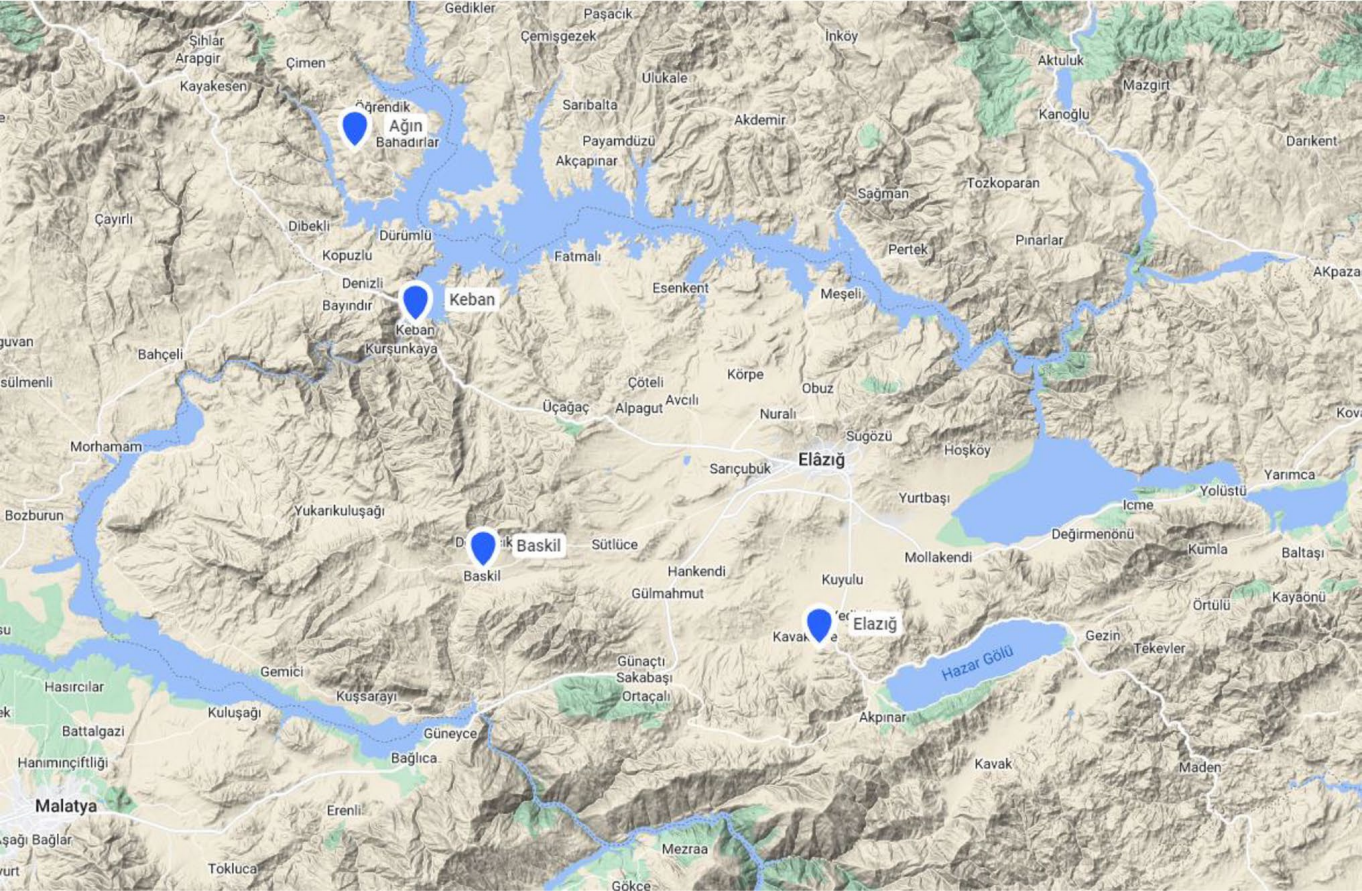
Study area

Relative humidity (average, maximum, and minimum) and temperature (average, maximum, and minimum), precipitation data, solar hour, evapotranspiration, and mean wind speed data in daily format were obtained from the General Directorate of Meteorology. In this study, data deficiencies were completed using the homogeneity method, and then the time series were turned into monthly format. The location of the study area is shown in Fig. 1. Elazig has a severe winter and hot and dry summers. Elazig has a continental climate with cold and rainy winters and hot and dry summers. Forecasting ET values is crucial since Elazig’s economic income is based on animal husbandry and agricultural production (especially apricots, sugar beets, and grapes). The proposed model used precipitation, temperature, relative humidity, solar hour, and wind speed to compute evapotranspiration.

## Material and method

The Levenberg Marquardt (LM) was applied to optimize the network weights since this algorithm is fast and powerful. Evapotranspiration values were estimated by ANN model development with the Levenberg–Marquardt method using temperature (min, mean, and max), relative humidity (min, mean, and max), solar hour, mean wind speed, and rainfall for four stations (Elazig, Agin, Keban, Baskil) with the range of 1980–2022. The findings obtained from the ANN-LM algorithm were compared with the Hargreaves method commonly used to calculate evapotranspiration in the literature.

### Hargreaves method

Jensen et al. (1997) and Zhai et al. (2010) stated that the Hargreaves equation (Hargreaves and Samani 1985) provides a simple analytical expression and is considered to be one of the most precise equations for computing ET:

1 $$ET=0.0023{R}_{a}\left(\frac{{T}_{Min}+{T}_{Max}}{2}+17.8\right)\sqrt{{T}_{Min}+{T}_{Max}}$$

in which *T*_*max*_ and *T*_*min*_ are temperatures of the minimum and maximum (°C), respectively. *R*_*a*_ is extraterrestrial radiation (mm/day), and ET is reference evapotranspiration (mm/day).

### Artificial Neural Network (ANN)

The theory of artificial neural network (ANN) succeeds as a parallel model of distributed networks with biological foundations in the human brain’s learning process. Numerous ANN applications exist in data analysis, adaptive control, and pattern recognition (Zhang and Friedrich 2003). The ANN is a computational system for simulating how the human brain handles, learns, and processes information. ANNs are a key type of artificial intelligence application capable of addressing complex issues challenged by human and statistical standards (Tiyasha et al. 2020). Moreover, ANN usually possesses powerful capabilities to approximate functions which are not known or to estimate values in the future based on the time series data that are potentially noisy (Hameed et al. 2017; Bisoyi et al. 2019; AlOmar et al. 2020). An ANN’s structure is composed of some simple components running in parallel. Identifying the ANN’s function, like natural processing, essentially depends on the links between elements (Yaseen et al. 2016). Generally, ANN has three layers: input and output (Bhagat et al. 2020).

A hidden layer is a key element of an ANN as it is placed among the output and the input layers, from which the neurons take several weighted inputs and thus produce output by implementing a particular automation function (Bhagat et al. 2020).

### Levenberg–Marquardt method

It is a modified implementation of Newton’s method for how to minimize functions described as the sum of the squares of other nonlinear functions (Hagan et al. 2003; Singh et al. 2007). The performance index applies to neural network training, defined as the mean squared error.

The Newton method for the optimization of a performance index *F*_*x*_ is (Singh et al. 2007):

2 $${x}_{k+1}={x}_{k}-{A}_{k}^{-1}{g}_{k}$$

in which ${g}_{k}={\left.\nabla F(x)\right|}_{x={x}_{k}}$ and ${A}_{k}={\left.{\nabla }^{2}F(x)\right|}_{x={x}_{k}}$

If the sum of squares function represents with $F(x)$:

3 $$F\left(x\right)=\sum\nolimits_{i=1}^{N}{\nu }_{i}^{2}\left(x\right)={\nu }^{T}(x)\nu (x)$$

4 $${[\nabla F(x)]}_{j}=2\sum\nolimits_{i=1}^{N}{\nu }_{i}\left(x\right)\frac{\partial {\nu }_{i}\left(x\right)}{\partial {x}_{j}}$$

The next value of ${x}_{k}$ is computed in the following:

5 $${x}_{k+1}={x}_{k}-{\left[2{J}^{T}\left({x}_{k}\right)J\left({x}_{k}\right)\right]}^{-1}{J}^{T}({x}_{k}) \nu ({x}_{k})$$

The Gauss–Newton method is presented in Eq. 5. It does not need the second derivative calculation, which is superior to the standard Newton method. It uses the following modification to the Hessian matrix approximation (Singh et al. 2007):

6 $$G=H+\mu I$$

7a $${x}_{k+1}={x}_{k}-{\left[{J}^{T}\left({x}_{k}\right)J\left({x}_{k}\right)+{\mu }_{k}I\right]}^{-1}{J}^{T}({x}_{k}) \nu ({x}_{k})$$

or

7b $$\Delta {x}_{k}={-\left[{J}^{T}\left({x}_{k}\right)J\left({x}_{k}\right)+{\mu }_{k}I\right]}^{-1}{J}^{T}({x}_{k}) \nu ({x}_{k})$$

It becomes beneficial because it approaches the algorithm of steepest descent with a low learning rate as ${\mu }_{k}$ increases (Singh et al. 2007).

As ${\mu }_{k}$ increases, it approaches the steepest descent algorithm with a low learning rate.

8 $${x}_{k+1}={x}_{k}-\frac{1}{{\mu }_{k}}{J}^{T}\left({x}_{k}\right)\nu \left({x}_{k}\right)={x}_{k}-\frac{1}{{2\mu }_{k}}\nabla F(x)$$

This LM algorithm is applied to the problem of multilayer network training (Hagan et al. 2003). Its performance index for multilayer network training is the mean squared error. When each objective is realized with equal probability, the mean squared error is equal to the sum of the squared error on *Q* targets in the training set (Singh et al. 2007):

9 $$F\left(x\right)=\sum\nolimits_{q=1}^{Q}{\left({t}_{q}-{a}_{q}\right)}^{T}\left({t}_{q}-{a}_{q}\right)=\sum\nolimits_{q=1}^{Q}{\left({e}_{q}\right)}^{T}{e}_{q}=\sum\nolimits_{q=1}^{Q}\sum\nolimits_{j=1}^{{s}^{M}}{\left({e}_{j,q}\right)}^{2}=\sum\nolimits_{i=1}^{N}({{\nu }_{i})}^{2}$$

${e}_{j,q}$ is the error of *j*th element for *q*th input.

The calculation of the Jacobian matrix is the key step in LM. To perform this calculation, a variation of the back-propagation algorithm is applied. The matrix is constructed by calculating the derivatives of the errors rather than the derivatives of the squared errors:

10 $${V}^{T}=\left[{\nu }_{1}, {v}_{2}, \dots , {v}_{N}\right]=[{e}_{1, 1}, {e}_{2, 1}, \dots ,{e}_{{s}^{M},1}, {e}_{\mathrm{1,2}}, \dots , {e}_{{s}^{M},Q}]$$

The vector parameter is as follows:

11 $${X}^{T}=\left[{x}_{1}, {x}_{2}, \dots , {x}_{N}\right]=[{w}_{1, 1}^{1}, {w}_{2, 1}^{1}, \dots ,{w}_{{s}^{1},R}^{1},{b}_{1}^{1}, \dots , {b}_{{s}^{1}}^{1}, {w}_{\mathrm{1,1}}^{2},\dots , {b}_{{s}^{M}}^{M}]$$

Standard back propagation is computed as follows (Singh et al. 2007):

12 $$\frac{\partial F\left(x\right)}{\partial {x}_{l}}=\frac{\partial {e}_{q}^{T}}{\partial {x}_{l}}$$

The Jacobian matrix elements for the LM algorithm need to be calculated as follows:

13 $${\left[J\right]}_{h,l}=\frac{\partial {v}_{h}}{\partial {x}_{l}}=\frac{\partial {e}_{k,q}}{\partial {x}_{l}}$$

Jacobian matrix elements can be calculated in the following:

14 $${\left[J\right]}_{h,l}=\frac{\partial {v}_{h}}{\partial {x}_{l}}=\frac{\partial {e}_{k,q}}{\partial {w}_{i,j}^{m}}=\frac{\partial {e}_{k,q}}{\partial {w}_{ii,q}^{m}}\frac{\partial {n}_{i,q}^{m}}{\partial {w}_{i,j}^{m}}={s}_{i,h}^{\sim m}\frac{\partial {n}_{i,q}^{m}}{\partial {w}_{i,j}^{m}}={s}_{i,h}^{\sim m}{a}_{j, q}^{m-1}$$

Or if *x*_*1*_ refers to a bias:

$$({n}_{i}^{m}=\sum_{j=1}^{{s}^{m-1}}{w}_{i,j}^{m}{a}_{j}^{m-1}+{b}_{i}^{m},$$ $$\frac{\partial {n}_{i}^{m}}{\partial {w}_{i,j}^{m}}={a}_{j}^{m-1}$$ and $$\frac{\partial {n}_{i}^{m}}{\partial {b}_{i}^{m}}=1$$

15 $${\left[J\right]}_{h,l}=\frac{\partial {v}_{h}}{\partial {x}_{l}}=\frac{\partial {e}_{k,q}}{\partial {b}_{i}^{m}}=\frac{\partial {e}_{k,q}}{\partial {n}_{i,q}^{m}}\frac{\partial {n}_{i,q}^{m}}{\partial {b}_{i}^{m}}=\frac{\partial {e}_{k,q}}{\partial {n}_{i,q}^{m}}\frac{\partial {n}_{i,q}^{m}}{\partial {b}_{i}^{m}}\frac{\partial {n}_{i,q}^{m}}{\partial {b}_{i}^{m}}={s}_{i,h}^{\sim m}$$

By modifying the last layer, Marquardt sensitivities can be derived via iteration relations (Eq. (10)). Corresponding Marquardt sensitivities at the last layer is below:

16 $$s_{i,h}^{\sim M}=\frac{\partial v_h}{\partial n_{i,q}^M}=\frac{\partial e_{k,q}}{\partial n_{i,q}^M}=\frac{\partial(t_{k,q}-a_{k,q}^M)}{\partial n_{i,q}^M}=\frac{\partial a_{k,q}^M}{\partial n_{k,q}^M}=\left\{\begin{array}{l}-f^M\left(n_{i,q}^M\right)\;for\;i=k\\0\;for\;i\neq k\end{array}\right.$$

If the *P*_*q*_ is implemented and *a*_*q*_^*M*^ (corresponding network output) is calculated, back-propagation of the LM starts:

17 $${S}_{q}^{\sim M}=-{F}^{M}({n}_{q}^{M})$$

in which Eq. (9) depicts the $-{F}^{M}({n}_{q}^{M})$ term. To produce a row of the Jacobian matrix, Eq. (10) is used, where each column of the matrix (${S}_{q}^{\sim M}$) must be back-propagated through the network. We can also back-propagate the column together as follows:

18 $${S}_{q}^{\sim m}={F}^{m}\left({n}_{q}^{m}\right){\left({w}^{m+1}\right)}^{T}{S}_{q}^{\sim m+1}$$

The overall Marquardt sensitivity matrix is generated by incrementing the calculated matrix for each layer and input:

19 $${S}^{\sim m}=[{S}_{1}^{\sim m}\left|{S}_{2}^{\sim m}\dots \left|{S}_{Q}^{\sim m}]\right.\right.$$

The sensitivity vectors of the *S*^*M*^ will be back-propagated for each input provided to the network. This is due to the fact that the derivative of each error is computed rather than the derivative of the sum of the squares of the errors. The *S*^*M*^ errors will exist for each input introduced into the network (Singh et al. 2007).

### ANN model development with the Levenberg–Marquardt method

**Fig. 2:**
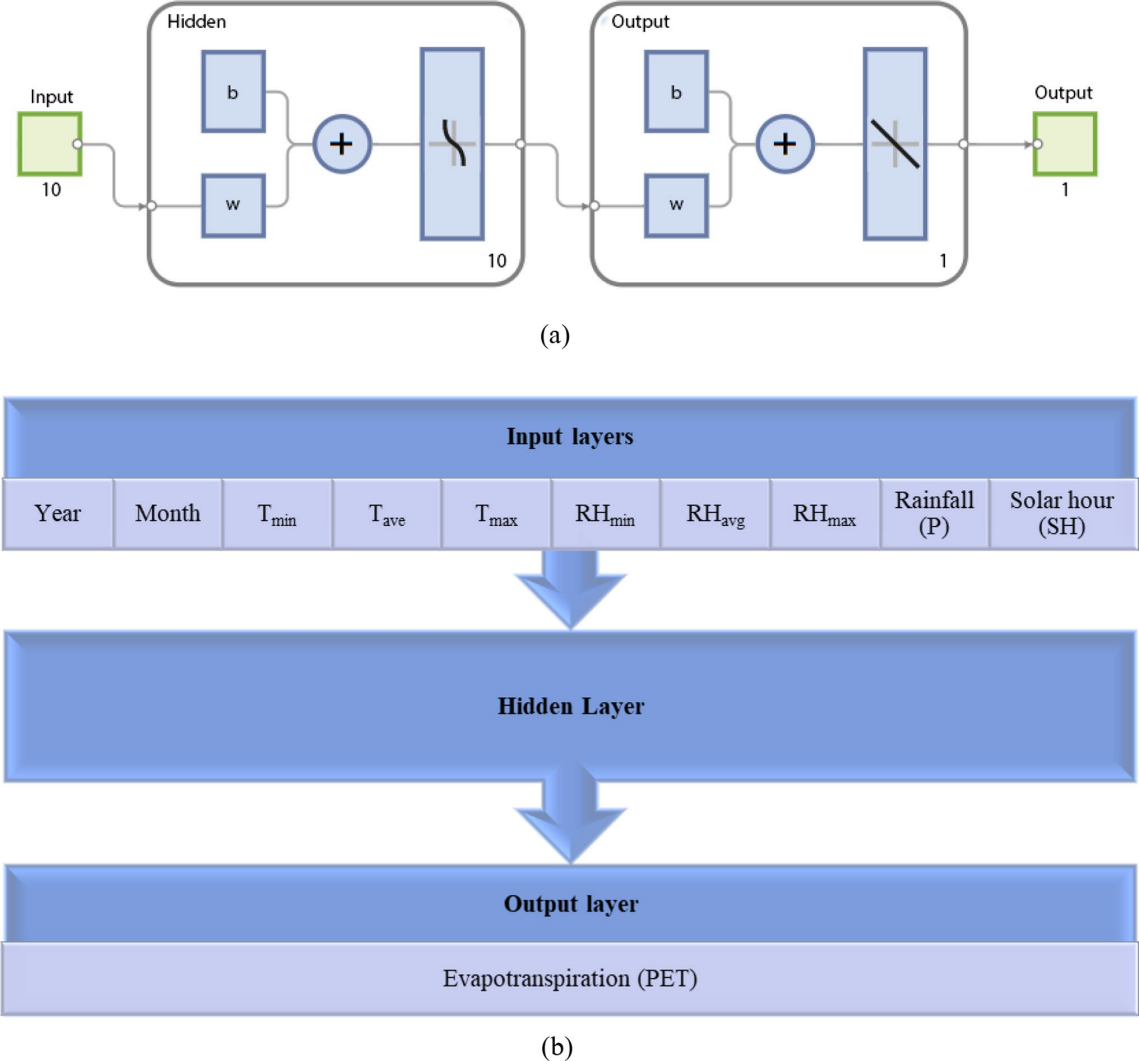
The effectiveness of the training process of ANN architectures for all locations

An ANN based on multilayer perceptron (MLP) architecture was developed to evaluate evapotranspiration values in the geographical regions of Elazig, Keban, Baskil, and Agin located in Turkey. Multi-layer perceptron (MLP) networks are a type of ANN characterized by their interconnected layers. These models are renowned for their robust systematic architecture, which enables them to achieve high levels of predictive accuracy. The MLP network comprises three layers, namely, the input, hidden, and output layers, arranged in a specific structural configuration. The input layer of the developed MLP incorporates various input parameters, including year, month, minimum humidity, maximum humidity, average humidity, wind speed, solar hour, rainfall, minimum temperature, maximum temperature, and average temperature. According to the network model, the subsequent layer following the input layer is referred to as the hidden layer, and it is mandatory for every MLP to possess at least one hidden layer. A challenge encountered in the advancement of MLPs pertains to the absence of a standardized mechanism for computing the noteworthy numerical component, commonly referred to as the neuron, which is identified within the hidden layer. The prevalent approach for determining the number of neurons in the hidden layer involves assessing the efficacy of network models generated with varying neuron quantities. The present study examines the performance of MLP networks with different numbers of neurons in a developed network structure. Based on the performance analyses, the MLP models with 16, 20, 12, and 16 neurons in the hidden layer are chosen for the Elazig, Keban, Baskil, and Agin locations, respectively. The evapotranspiration parameter was estimated in the output layer, which immediately follows the hidden layer. Figure 2 presents the symbolic configuration scheme and the fundamental architecture of the MLP that was generated. The ANN was constructed and assessed using a dataset comprising 516 data points. The dataset was divided into three subsets, with 362 data points allocated for training, 77 for validation, and 77 for testing. The LM-type ANN training algorithm is a commonly utilized method due to its superior learning capabilities and is therefore the preferred choice for training ANNs. The multilayer perceptron (MLP) network has been designed to estimate evapotranspiration for specific locations by utilizing the Tan-Sig and Purelin transfer functions in the hidden and output layers. The mathematical expressions for the transfer functions are as follows (Vafaei et al. 2017):

20 $$f(x)=\frac{1}{1+{\text{exp}}(-x)}$$

21 $$purelin(x)=x$$

### Performance indices

The evaluation of the estimation performance of the designed ANN involves the selection of commonly utilized parameters such as the coefficient of correlation (*R*) and mean squared error (MSE). In addition, the ANN model calculates the evapotranspiration parameter alongside their respective margin of deviation (MoD) values, which indicate the proportional deviation from the target values. The subsequent discourse presents the statistical formulas employed in calculating the performance metrics as given by Ocal et al. (2021):

22 $$MSE=\frac{1}{N}\sum\limits_{i=1}^{N}{\left({X}_{{\text{exp}}(i)}-{X}_{ANN(i)}\right)}^{2}$$

23 $$R=\sqrt{1-\frac{\sum\limits_{i=1}^{N}{\left({X}_{{\text{exp}}(i)}-{X}_{ANN(i)}\right)}^{2}}{\sum\limits_{i=1}^{N}{\left({X}_{{\text{exp}}(i)}\right)}^{2}}}$$

24 $$MoD(\%)=\left[\frac{{X}_{{\text{exp}}}-{X}_{ANN}}{{X}_{{\text{exp}}}}\right]\times 100$$

25 $$MAPE=\frac{\sum\limits_{i=1}^{n}\left|\frac{{A}_{i}-{F}_{i}}{{A}_{i}}\right|}{n}\times 100$$

## Results and discussion

**Fig. 3:**
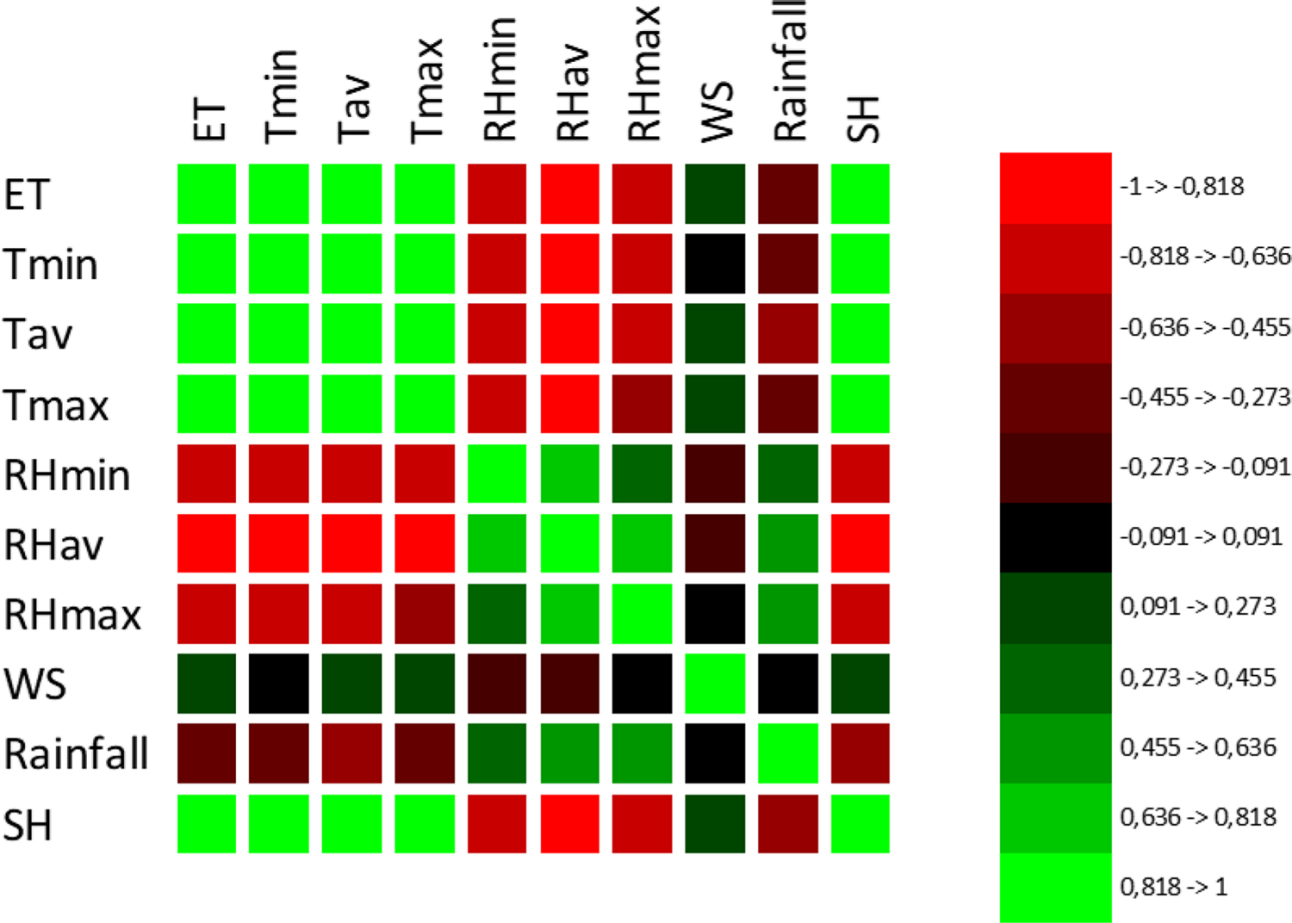
Correlation map of evapotranspiration

The present study presents the prediction results of evapotranspiration using the minimum, average, and maximum temperatures (*T*_*min*_, *T*_*av*_, *T*_*max*_); minimum, average, and maximum relative humidities (*RH*_*min*_, *RH*_*av*_, *RH*_*max*_); wind speed (WS); rainfall (P); and solar hour (SH) based on ANN-Levenberg Marquardt algorithm. A correlation test was applied to determine which parameters are effective on the evapotranspiration before setting the model. The degree of effects of the input parameters in the prediction of ET at a 95% confidence interval was determined via correlation analysis. The bold ones in Table 1 show significant correlation coefficients at the 95% confidence interval. It was determined that the minimum, average, maximum temperatures (*T*_*min*_, *T*_*av*_, *T*_*max*_); minimum, average, maximum relative humidities (*RH*_*min*_, *RH*_*av*_, *RH*_*max*_); wind speed (WS); rainfall (P); and solar hour (SH) are adequate on the prediction of evapotranspiration. The correlation map is shown in Fig. 3. As seen in Fig. 3, *T*_*min*_, *T*_*av*_, *T*_*max*_, WS, and SH have a positive correlation on the PET, while *P*, *RH*_*min*_, *RH*_*av*_, and *RH*_*max*_ have a negative correlation.

**Table 1:** The results of the Correlation analysis

Variables	*ET*	*T*_*min*_	*T*_*av*_	*T*_*max*_	*RH*_*min*_	*RH*_*av*_	*RH*_*max*_	WS	Rainfall	SH
*ET*	**1**	**0.892**	**0.942**	**0.941**	** − 0.709**	** − 0.874**	** − 0.644**	**0.251**	** − 0.437**	**0.917**
*T*_*min*_	**0.892**	**1**	**0.974**	**0.933**	** − 0.696**	** − 0.862**	** − 0.690**	0.055	** − 0.448**	**0.875**
*T*_*av*_	**0.942**	**0.974**	**1**	**0.978**	** − 0.732**	** − 0.909**	** − 0.689**	**0.132**	** − 0.480**	**0.913**
*T*_*max*_	**0.941**	**0.933**	**0.978**	**1**	** − 0.756**	** − 0.901**	** − 0.624**	**0.164**	** − 0.446**	**0.909**
*RH*_*min*_	** − 0.709**	** − 0.696**	** − 0.732**	** − 0.756**	**1**	**0.803**	**0.428**	** − 0.096**	**0.356**	** − 0.693**
*RH*_*av*_	** − 0.874**	** − 0.862**	** − 0.909**	** − 0.901**	**0.803**	**1**	**0.718**	** − 0.154**	**0.617**	** − 0.875**
*RH*_*max*_	** − 0.644**	** − 0.690**	** − 0.689**	** − 0.624**	**0.428**	**0.718**	**1**	− 0.077	**0.546**	** − 0.645**
WS	**0.251**	0.055	**0.132**	**0.164**	** − 0.096**	** − 0.154**	− 0.077	**1**	− 0.071	**0.217**
Rainfall	** − 0.437**	** − 0.448**	** − 0.480**	** − 0.446**	**0.356**	**0.617**	**0.546**	− 0.071	**1**	** − 0.510**
SH	**0.917**	**0.875**	**0.913**	**0.909**	** − 0.693**	** − 0.875**	** − 0.645**	**0.217**	** − 0.510**	**1**

Values in bold are different from 0 with a significance level alpha = 0.05

ANNs are a crucial tool in predicting evapotranspiration due to their ability to capture intricate and nonlinear associations between input and output variables. This is a challenging feat to accomplish using conventional modeling techniques. Predicting evapotranspiration is a complex process involving considering various parameters, which may exhibit nonlinear interactions. These parameters are contingent upon multiple factors, including but not limited to the year, month, evaporation rate, minimum, maximum, and average humidity levels, solar hour, wind speed, rainfall, and minimum, maximum, and average temperatures. ANNs have the potential to address the limitations of conventional modeling techniques, particularly in accurately capturing intricate nonlinear associations between input and output variables. ANNs are a suitable tool for modeling intricate two-phase flow systems because they effectively manage voluminous datasets encompassing a diverse range of input variables. One additional benefit of utilizing ANNs in evapotranspiration forecasting is their ability to acquire knowledge from empirical observations. An ANN can be trained to accurately identify the true correlations between the input and output variables by utilizing empirical data. The network can subsequently utilize the acquired knowledge to predict novel data. This can prove to be particularly advantageous as conventional models may not accurately predict the behavior of evapotranspiration under diverse operational circumstances.

ANNs have the capability to employ machine learning techniques to facilitate the process of determining the appropriate transformation of anticipated inputs into corresponding outputs. ANNs research to establish the relationship between the data and outcomes of the training dataset. The assessment of the dataset’s efficacy is conducted through the utilization of an evaluation sample. Time proof is utilized as an estimation for the duration of ANN training. ANNs exhibit suboptimal performance on the test dataset when the training dataset manifests over-fitting, leading to prolonged learning. The optimal termination point of the training phase can be determined by utilizing a confirmation dataset. The performance of ANNs is evaluated based on the accomplishments they are capable of predicting. In function estimation, open sources facilitate the integration of diverse types and topologies of artificial neural networks. Several prevalent techniques involve the amalgamation of fuzzy logic decision systems with ANNs. These techniques encompass multi-layer perceptrons (MLPs), radial basis functions (RBFs), generalized regression neural networks (GRNNs), and the artificial neural fuzzy inference system (ANFIS).

**Fig. 4:**
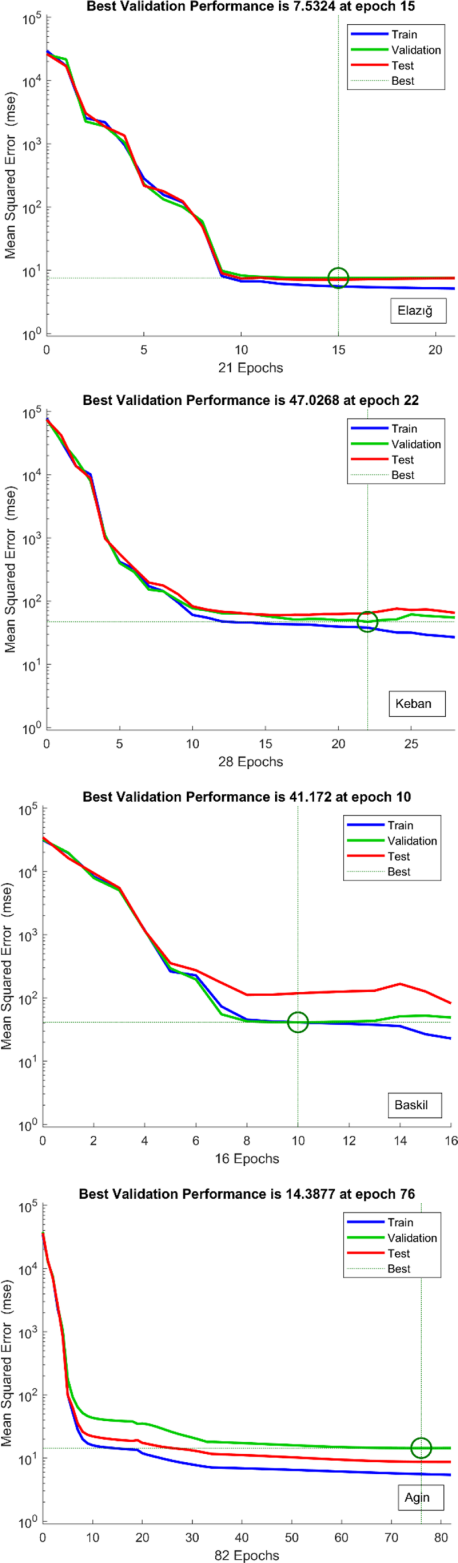
The training performance of the ANN model in model for all locations

The initial stage in evaluating the predictive capability of the ANN architecture is to verify the successful completion of the network’s training and learning phases. During the training phase of MLP networks, the information transmitted from the input layer to the output layer is subsequently fed back to the input layer to minimize errors. This iterative process is commonly known as an “epoch.” It is anticipated that at the end of each epoch, the disparity between the target and prediction data will diminish, decreasing the mean squared errors (MSEs). Upon reviewing the training performance graph depicted in Fig. 4, it can be observed that the mean squared errors (MSEs) exhibit high values during the initial stages of the multilayer perceptron (MLP) algorithm’s training process. However, as the training progresses through each epoch, the MSEs gradually decrease towards the outcome. The training phase of the ANN model is deemed to be finished when the mean squared errors (MSEs) acquired for both the training and testing procedures converge towards the optimal value. The results obtained from the training performance data suggest that the ANN training was effectively executed. The second figure illustrates the difference between the pressure drop obtained from experimental data and the results produced by the ANN in relation to the epoch versus MSE. As depicted in Fig. 4, the MSE metric, which demonstrated a high value in its initial phase, displays a decreasing pattern in the following periods. Based on the available data, it appears that the training process of the artificial neural network was executed optimally. This is evidenced by the attainment of the most favorable outcome, with a minimal MSE value of 0.2041, − 0.468, − 1.869, and 0.2863, respectively, after 21, 28, 16, and 82 iterations for the regions of Elazig, Keban, Baskil, and Agin.

**Fig. 5:**
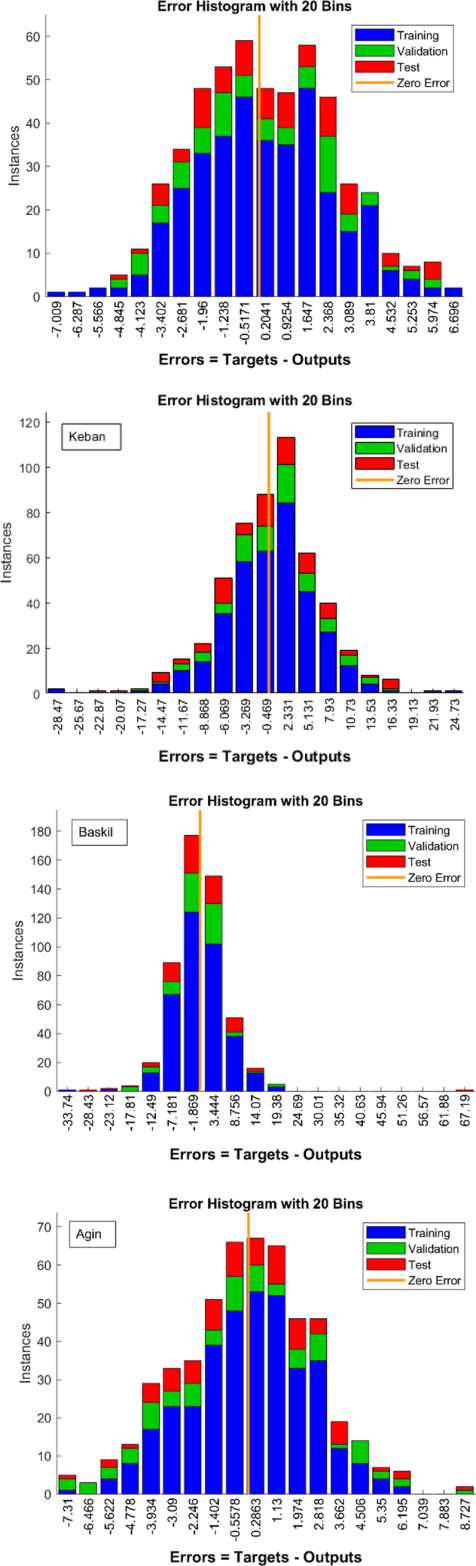
Error histogram of the ANN in the model for all locations

When evaluating the effectiveness of an ANN, it is crucial to examine the histogram plot of the error. The histogram depicted in Fig. 5 pertains to the ANN specifically developed for Elazig, Keban, Baskil, and Agin regions. The histogram graph depicting errors presents all the data gathered during the artificial neural network’s various training, testing, and validation stages. Figure 5 demonstrates that the data utilized for training, testing, and validation was collected near the axis of zero error. The distribution presented suggests that the artificial neural network model constructed demonstrates a reasonable degree of imprecision in its prediction ability.

**Fig. 6:**
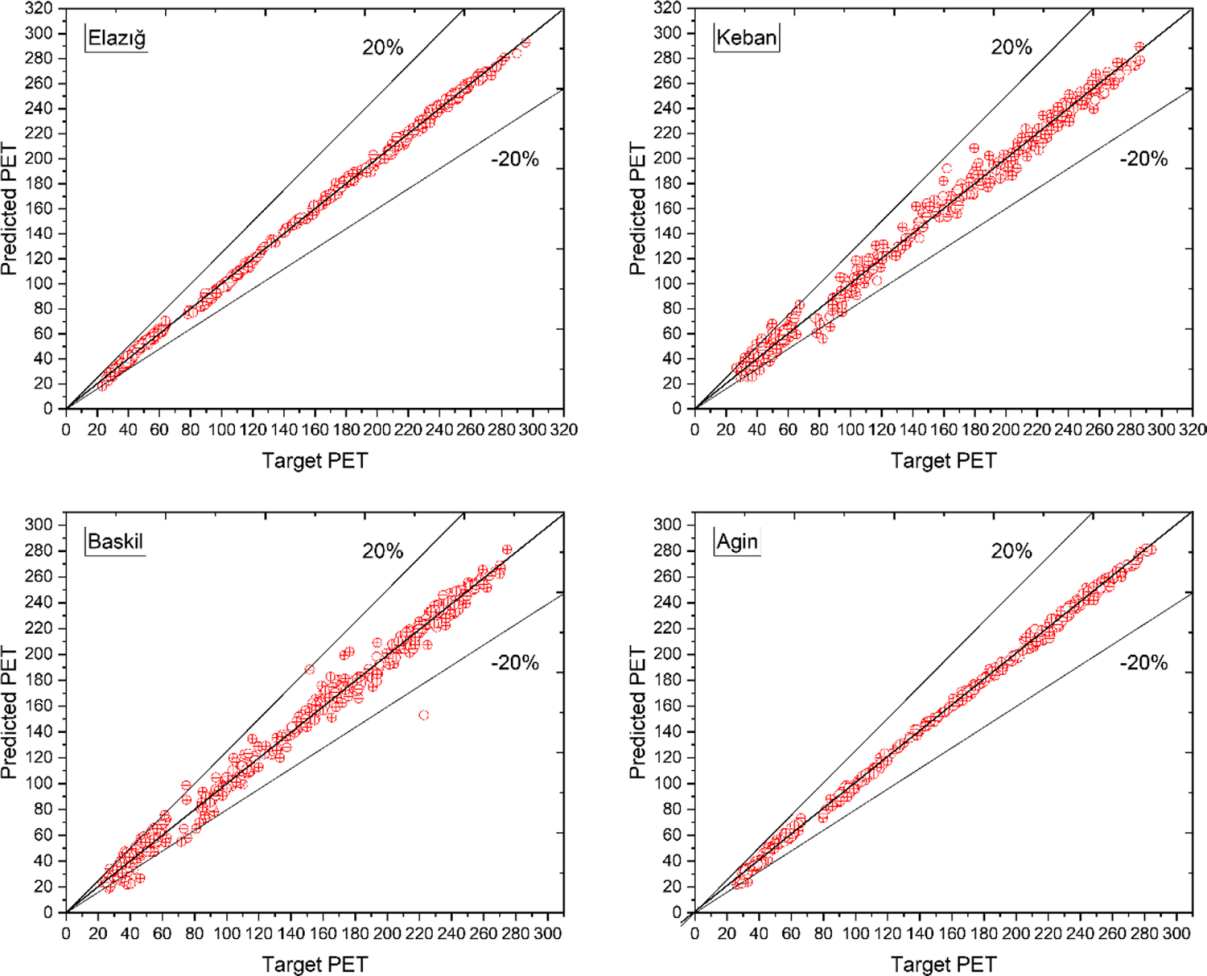
The predicted and target PET values of related locations

The horizontal axis in Fig. 6 represents the empirical data, which serves as the objective data, while the vertical axis displays the approximations produced by the ANN. Upon analysis of the data, it is evident that each data point is located in close proximity to the zero-error line. The results suggest that the designed ANN structure may provide precise predictions. The ANN model produced *R*-values of 0.9995, 0.9948, 0.9898, and 0.9994 for the Elazig, Keban, Baskil, and Agin sites, respectively. After conducting a thorough analysis of the MSE and MoD for each output presented in Table 2, it is apparent that the ANN has been constructed with a noteworthy ability to generate accurate predictions. The study obtained extensive numerical data, conducted a thorough evaluation of performance parameters, and accurately aligned the target data with the output of the ANN model. The results unequivocally indicate that the optimized ANN is capable of producing estimations with minimal and acceptable variances and exceptional precision.

**Table 2:** Parameters of the ANN models

		Elazig	Keban	Baskil	Agin
Number of data		516	516	516	516
Input No		10	10	10	10
Neuron No		10	10	10	10
MSE		0.2041	− 0.469	− 1.869	0.2863
R		0.99985	0.9948	0.9898	0.9994
MoD (%)	Min	− 0.1032	− 0.3703	− 0.3186	− 0.1219
	Max	0.2287	0.3184	0.4452	0.2784
	Ave	0.0027	− 0.0052	− 0.0018	6.08E-05

**Fig. 7:**
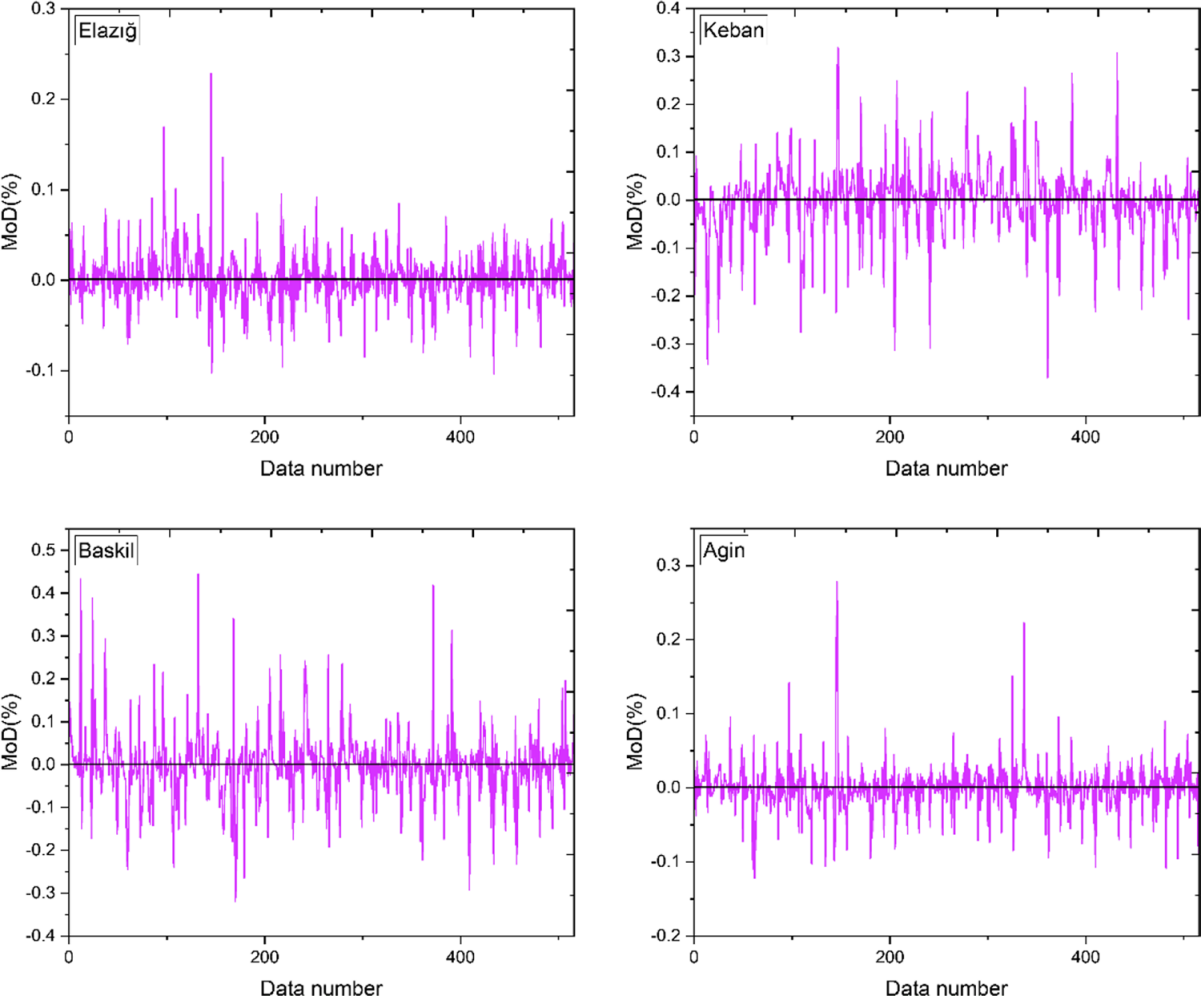
The calculated MoD values for each data point for all locations

Figure 7 visually depicts the MoDs determined for each data point. After careful examination of the calculated data points, it is evident that they demonstrate a propensity to cluster in close proximity to the line of zero error. The proximity of the MoD to zero and their relatively small outcomes imply that the difference between the ANN outputs and the target data is not significant and satisfactory. Figure 7 displays fluctuations of approximately ± 0.3%, ± 0.4%, ± 0.5%, and ± 0.3% for the condensation and evaporation conditions of the models, respectively, for the locations of Elazig, Keban, Baskil, and Agin.

**Fig. 8:**
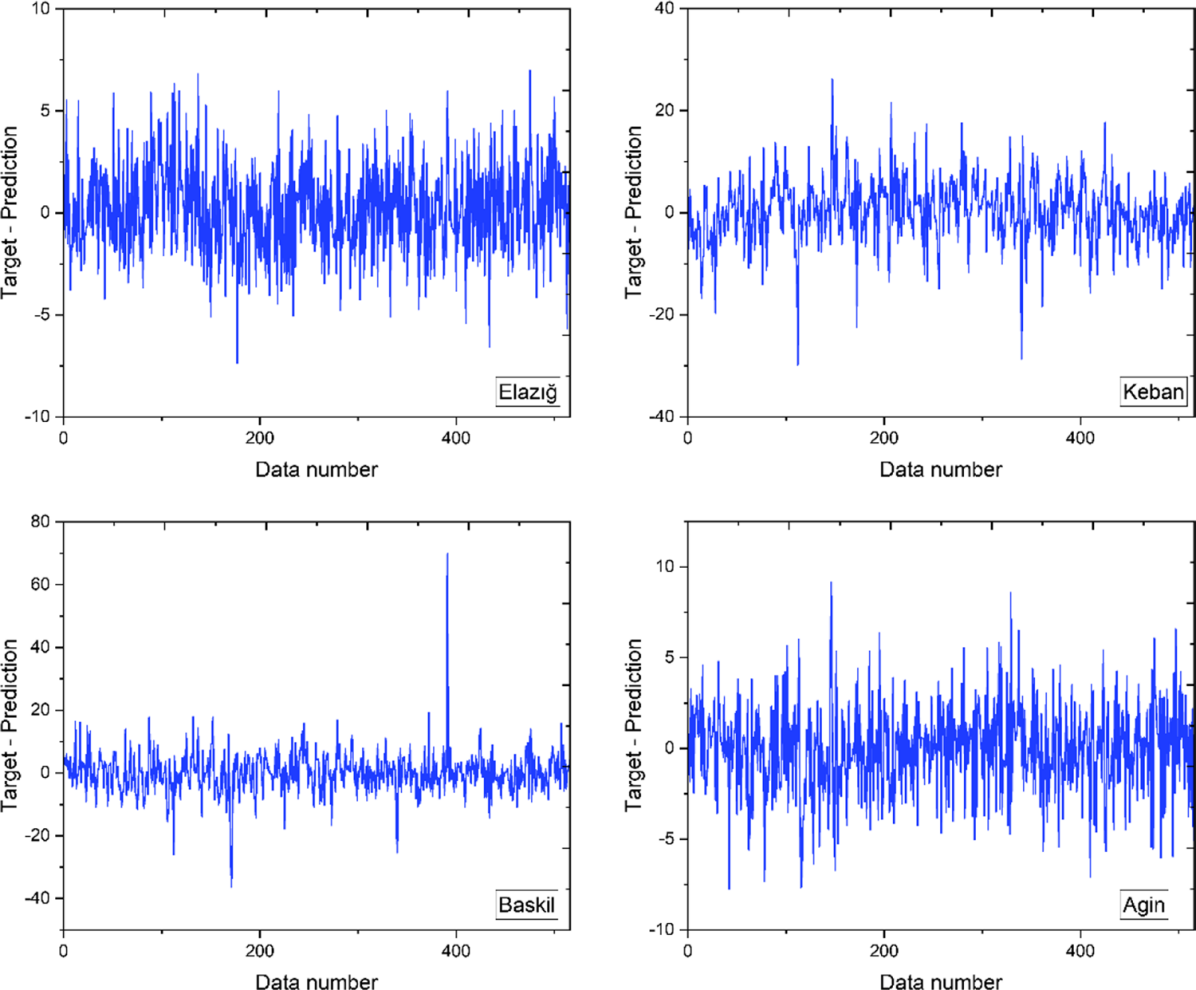
The differences between target values and ANN outputs for all locations

Figure 8 illustrates the discrepancies between the prognostic values generated by the ANN and the target values assigned to each datum, providing a comprehensive evaluation of the ANN’s prognostic capacity. After analyzing the aforementioned figure, it can be observed that the differences between the values obtained for each point are relatively insignificant. Therefore, it is reasonable to infer that the aforementioned ANN model has the potential to produce predictions with a significantly reduced degree of inaccuracy. According to the analysis findings, Elazig exhibits a maximum discrepancy value of 7 and a minimum value of − 7.4. The maximum disparity value documented for Keban is − 29.8, whereas the minimum is 26.2. The maximum disparity value documented for Baskil is − 36.4, whereas the minimum value is 69.9. The maximum disparity value documented for Agin is − 7.8, whereas the minimum is 9.2. The disparities between the expected and intended values for each datum were assessed, and the resultant inconsistencies for each point were graphically depicted in Fig. 8. In addition to considering proportional errors, analyzing the differences between the predicted values obtained for each data point and the actual data can provide valuable information regarding the model’s predictive accuracy. The discrepancies between the designated target values for each data point and the corresponding outputs produced by the ANN were calculated to achieve this goal.

**Fig. 9:**
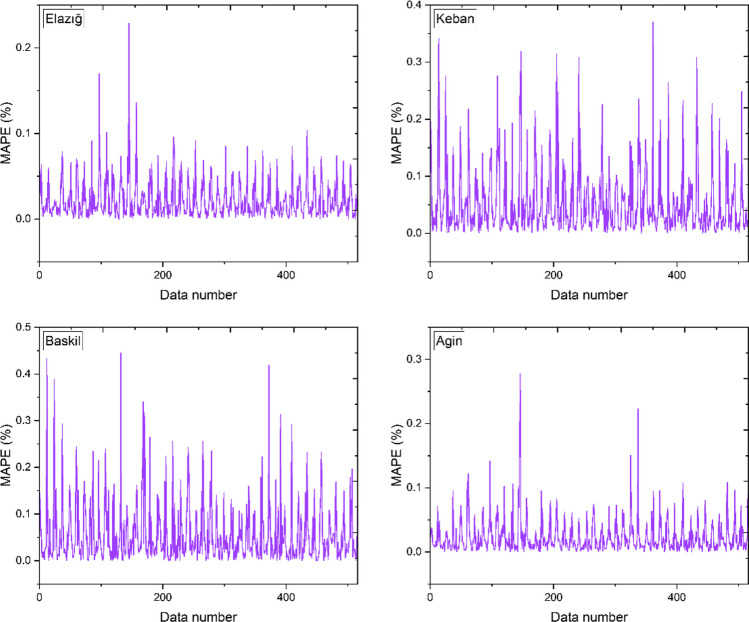
Mean absolute percentage results for all locations

Figure 9 displays the MAPE values pertaining to Elazig, Keban, Baskil, and Agin. The investigation calculated the minimum and maximum mean absolute percentage error (MAPE) values pertaining to Elazig and Keban. The MAPE values for Elazig and Keban were observed to vary between 0 and 0.2288 and 0.0001 and 0.3703, respectively. The maximum and minimum MAPE values for Baskil were identified as 0 and 0.4453, respectively. Likewise, the highest and lowest MAPE values for Agin were determined to be 0 and 0.2784, correspondingly.

**Fig. 10:**
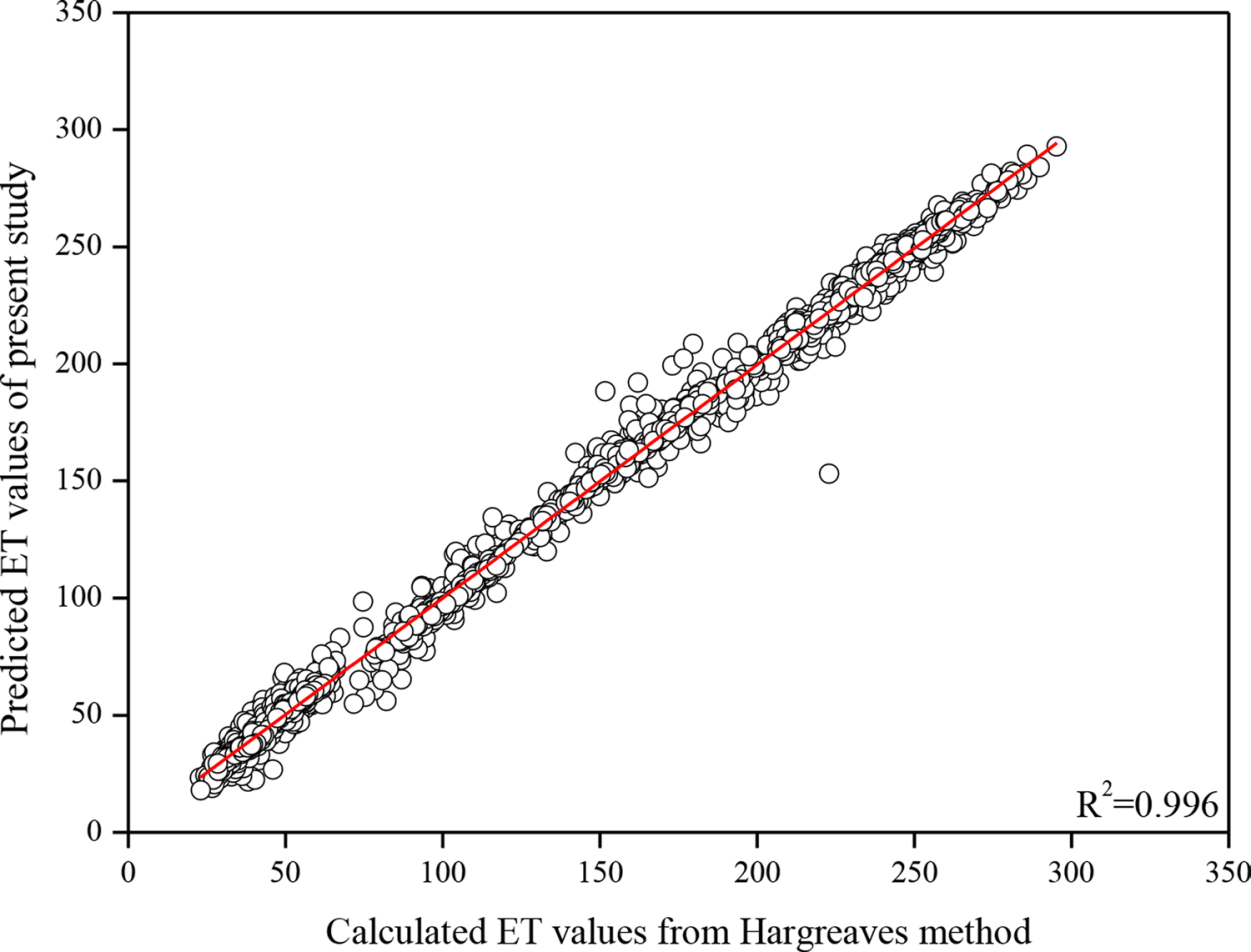
The comparison of predicted ET values of the present study and calculated ET values from the Hargreaves method

The comparison of predicted ET values of the present study by ANN utilizing the Levenberg–Marquardt method and calculated ET values from the Hargreaves method is shown in Fig. 10. Especially in regions where ET data is unavailable, the Hargreaves method is one of the most widely used methods for calculating ET values. As seen in Fig. 10, the results are consistent (*R*^2^ = 0.996). Therefore, it can be stated the results of the study are reliable.

The examination of MoDs provides empirical evidence that supports the proficiency of the constructed ANNs in forecasting with minimal discrepancies and maximal precision. The ANN models’ design and performance parameters are presented in Table 2.

## Discussion

Evapotranspiration is the water loss from soil and crop surfaces to the atmosphere through evaporation and transpiration. It is considered to be a critical contributor in water balance and irrigation management (Huo et al. 2012). Evapotranspiration can be directly measured using costly micrometeorological techniques based on methods of transferring water vapor mass flux and energy balance (Landeras et al. 2008). Nevertheless, it is less costly to apply mathematical models with measured meteorological parameters as independent variables for evapotranspiration prediction (Huo et al. 2012).

Several models for evapotranspiration estimation with limited meteorological input based on artificial neural networks are available in the literature. Kişi (2006) analyzed and discussed the performance of conjugate gradient (CG) and Levenberg–Marquardt (LM) algorithms in evapotranspiration estimation. Several combinations of solar hour, air and soil temperature, wind speed (WS), and relative humidity (RH) data were considered as input to artificial neural network (ANN) models, and how each of these variables affects evapotranspiration was evaluated. The ANN model results are compared with multiple linear regression (MLR) and Hargreaves and Penman’s empirical models. The results show that solely using only the WS as input is insufficient to model ET. However, it was found that the addition of WS to the input combinations improved the performance of the models, and the use of only air temperature in modeling ET resulted in weak predictions. It was also reported that removing soil temperature from the input combinations led to a better prediction of evapotranspiration. The best performance was obtained for the models with wind speed (WS), solar hour (SH), relative humidity (RH), and air temperature (T). The calibrated empirical models outperformed the literature’s widely used Penman and Hargreaves models. It was found that the ANN-LM outperformed the MLR, ANN-CGF, empirical Hargreaves, and Penman models based on comparisons (*R*^2^ = 0.983).

Kişi (2007) studied the prediction of evapotranspiration based on the ANN-LM algorithm. Different combinations (WS, T, RH, and SH) were used to predict evapotranspiration and evaluate the effect of these parameters on evapotranspiration in Los Angeles, USA, based on the ANN-LM. ANN-LM findings were compared with those obtained from the Penman, Turc, and Hargreaves empirical formulas. They determined that the Hargreaves method provided better results than the Turc and Penman methods in the prediction of evapotranspiration. They reported that the model with only SH provided better results than the model with the *WS* and *RH*, while the ANN model with only WS gave the worst results. It was found that the ANN model with *WS*, *SH*, *RH*, and *T* gave the best results among the other combinations (*R*^2^ = 0.963). So, it was reported that these parameters are necessary to predict ET.

Moreover, it was determined that *T* had more effect on the prediction of evapotranspiration than the *RH* and *WS*. It was reported that the model performance significantly increased when *T* was added as input. The authors highlighted that SH was the most critical parameter in the prediction of evapotranspiration.

Similarly, Huo et al. (2012) predicted evapotranspiration values based on different combinations via the ANN-LM algorithm in northwest China. They concluded that the model with *T*_*min*_, *T*_*max*_, *RH*, *SH*, and *WS* gave the best results among the tried models (*R*^2^ = 0.990–0.995). They determined that *RH* and *T* are the most critical parameters in evapotranspiration estimation. They highlighted that the ANN gave better results than MLRs and empirical formulas (Penman, Samani-Hargreaves and Priestley-Taylor).

Khedkar et al. (2019) conducted a study to predict evapotranspiration values using the ANN-LM technique. They used five models to obtain the best prediction results with ANN-LM. They reported that the model with *T*_*min*_, *T*_*max*_, *RH*_*min*_, *RH*_*max*_, *WS*, and *SH* outperformed the other models (0.996), while the model with pan evaporation had the worst results (*R* = 0.900). They obtained the results for the model with *T*_*min*_, *T*_*max*_, *RH*_*min*_, *RH*_*max*_, and SH (*R* = 0.976), the model with *T*_*min*_, *T*_*max*_, and *SH* (*R* = 0.969), and the model with *T*_*min*_ and *T*_*max*_ (*R* = 0.938). Moreover, they determined an upward trend in the values of *R* as the number of input parameters was increased in ANN models. They determined that ANN with five inputs had better results (*T*_*min*_, *T*_*max*_, *RH*_*min*_, *RH*_*max*_, and *SH*) than those of ANN with three or four inputs in the estimation of evapotranspiration in arid and semiarid regions of China.

Patle et al. (2020) studied the prediction of evapotranspiration using multiple linear regression (MLR) and ANN techniques in India with six input parameters (*T*_*max*_, *T*_*min*_, *RH*_*max*_, *RH*_*min*_, *SH*, and *WS*). The analysis showed that model performance is better as weather parameters combine. MLR has the highest *R*^2^ (0.62 and 0.90) for Sikkim’s training and testing phases, respectively. In contrast, the ANN model has the highest *R*^2^ (0.65 and 0.91) for Sikkim’s training and testing phases, respectively. Similar results were obtained for the Gangtok station. MLR model has the highest *R*^2^ (0.88 and 0.85) for Imphal’s training and testing phases. In contrast, the ANN model has the highest *R*^2^ (0.89 and 0.86) for the training and testing phases.

Achite et al. (2022) investigated the potential of GEP, FFNN, and RBFNN to predict daily evapotranspiration in Algeria using different meteorological variables. The results showed that both the neural network (FFNN and RBFNN) and GEP models make for optimal levels of agreement with the *ET*_0_ obtained by the FAO PM method. The results demonstrated that modeling evapotranspiration using the ANN technique gives better results than the GEP model. They stated that ANN and GEP models based on *T*_*ave*_, relative humidity, wind speed, and global radiation parameters are enough to obtain optimum estimation for daily evapotranspiration in the semi-arid region of Algeria.

Zhao et al. (2023) conducted a study to predict daily and monthly evapotranspiration values using gradient boosting decision tree (GBDT), random forest (RF), and extreme gradient boosting (XGBoost) in North China. They found that maximum, minimum, and average temperatures, as well as solar radiation, are effective in accurately predicting daily evapotranspiration values. They determined that the XGBoost model outperformed the GBDT and RF models in estimating daily and monthly evapotranspiration values.

Moreover, Wu et al. (2020) applied Extreme Gradient Boosting (XGB), Adaptive Boosting (ADA), GBDT, gradient boosting with categorical features support (CAT), and light gradient-boosting decision machine (LGB) to predict the daily evapotranspiration values in eastern China. They stated that CAT models have the highest accuracy (*R*^2^ = 0.8514).

Mohammadrezapour et al. (2019) applied the support vector machine (SVM), adaptive neuro-fuzzy inference system (ANFIS), and gene expression programming (GEP) to predict monthly evapotranspiration values in Iran. The input was selected as average temperature, relative humidity, wind speed, and solar hours. They found that the SVM model (*R*^2^ = 0.777–0.970) outperformed GEP (*R*^2^ = 0.775–0.984) and ANFIS (*R*^2^ = 0.18–0.889) models in the prediction of monthly evapotranspiration values.

This study estimated the evapotranspiration values of ANN model development with the Levenberg–Marquardt method in Elazig. Differently from the studies above, nine parameters (*T*_*min*_, *T*_*av*_, *T*_*max*_, *RH*_*min*_, *RH*_*av*_, *RH*_*max*_, *SH*, *WS*, and *P*) were used to predict evapotranspiration values. As seen in the studies above, the ANN-LM algorithm gives good results in predicting evapotranspiration (Kişi 2006, 2007; Huo et al. 2012; Khedkar et al. 2019). Kişi (2006, 2007), Huo et al. (2012), and Khedkar et al. (2019) used the ANN-LM algorithm to predict evapotranspiration. They generally used *T*_*min*_, *T*_*max*_, *RH*_*min*_, *RH*_*max*_, *SH*, and *WS*. Rainfall, average temperature, and relative humidity values were used in ANN-LM algorithm to predict evapotranspiration in addition to the above parameters in this study. Although a similar algorithm was used in the present study like Kişi (2006, 2007), Huo et al. (2012), and Khedkar et al. (2019), since more meteorological parameters were used (*T*_*min*_, *T*_*av*_, *T*_*max*_, *RH*_*min*_, *RH*_*av*_, *RH*_*max*_, *SH*, *WS*, and *P*), and better results were obtained (*R*^2^ = 0.9797–0.9997) when the present study was compared with the studies of Kişi (2006, 2007), Huo et al. (2012), and Khedkar et al. (2019). Moreover, the findings of the present study are generally similar and compatible with the studies of Kişi (2006, 2007), Huo et al. (2012), and Khedkar et al. (2019), and the evapotranspiration values of this study are compatible with the Hargreaves method like the studies Kişi (2006, 2007), Huo et al. (2012), and Khedkar et al. (2019). Moreover, better results were obtained since more input parameters and ANN-LM method were used (Table 2) when compared with the study of Patle et al (2020). For instance, *R*^2^ values are 0.9997 (Elazig), 0.9896 (Keban), 0.9797 (Baskil), and 0.9988 (Agin). All *R*^2^ values for stations are higher than that of Patle et al. (2020). However, Patle et al. (2020) obtained the highest *R*^2^ value for the Imphal station (0.91). As a result, it can be stated that ANN model development with Levenberg–Marquardt gives better results than ANN under the same conditions and is compatible with the literature. As a result, the study and the literature verified that the ANN is a valuable tool for modeling evapotranspiration. Several researchers reported that ANN prediction of evaporation is better than MLR prediction (Shirsath and Singh 2010; Ali and Saraf 2015; Shiri et al. 2015; Malik et al. 2018).

As a result, it was concluded that the ANN-LM technique could be successfully applied to modeling evapotranspiration from available climatic data.

## Conclusions

Using numerical methods enables weather scientists and affiliated communities to make predictions regarding evapotranspiration levels cost-effectively, compared to relying solely on experimental measurements. Consistent with the literature review findings, various machine learning models have been proposed for evapotranspiration prediction. However, the current study employs the Levenberg–Marquardt method. As a result, this research is expected to provide new perspectives on the progression of evapotranspiration. An ANN was employed to replicate a weather output attribute, specifically the occurrence of evapotranspiration. In order to train the developed ANN model, a total of 516 numerically recorded data sets were utilized. The allocation of datasets for the model’s training, validation, and testing has been established as follows: 362 datasets for training, 77 for validation, and 77 for testing. The LM-type artificial neural network method was employed to train the multilayer perceptron neural network, which incorporated a hidden layer comprising 10 neurons for all locations. The ANN model’s hidden and output layers employ Tan-Sig and Purelin transfer functions, respectively, while the input layer of the model is utilized. The present model incorporates a forecast for evapotranspiration. The efficacy of the ANN model has been evaluated concerning its training, learning, and predictive abilities. Nine parameters (min, mean and max temperature, min, mean and max humidity, solar hour, wind speed, and rainfall) were used to predict evapotranspiration values in the study. Although different parameters were used, similar results were obtained.

Moreover, the results obtained are compatible with that of the Hargreaves method, which is the most popular in the computation of evapotranspiration (*R*^2^ = 0.996). The performance analysis is conducted utilizing R and MoD features. The ANN models exhibit *R*-values of 0.9995, 0.9948, 0.9898, and 0.9994 for Elazig, Keban, Baskil, and Agin locations, respectively. The values for MSE were calculated as follows: 0.2041, − 0.468, − 1.869, and 0.2863, respectively. The mean MoD value computed for all output measurements was found to be near zero upon taking the average. The maximum MAPE values are calculated as 0.2288, 0.3703, 0.4453, and 0.2784 for the locations Elazig, Keban, Baskil, and Agin, respectively. ANNs have demonstrated significant utility as an engineering methodology for estimating ET values based on prevailing weather conditions. The ANN technique can be used in reservoir design and several hydrological analyses. Further studies using more data from various fields will be practical in evapotranspiration modeling.

## Data Availability

The datasets used and/or analyzed during the current study are available from the author on reasonable request.

## Nomenclature

ANFIS: Adaptive neuro-fuzzy inference system
ANN: Artificial neural network
ET: Evapotranspiration
GBDT: Gradient boost decision tree
MAPE: Mean absolute percentage errors.
ML: Machine learning
MLP: Multilayer perceptron
MLR: Multiple linear regression
MoD: Margin of deviations
MSE: Mean squared error.
SVM: Support vector machine
WNN: Wavelet neural networks
XGBoost: Extreme gradient boost

## Abbreviations

*R*: Coefficient of Correlation
*R*_*a*_: Extraterrestrial radiation
*T*: Temperature
